# A multi-gene expression profile panel for predicting liver metastasis: An algorithmic approach

**DOI:** 10.1371/journal.pone.0206400

**Published:** 2018-11-01

**Authors:** Kanisha Shah, Shanaya Patel, Sheefa Mirza, Rakesh M. Rawal

**Affiliations:** Division of Medicinal Chemistry & Pharmacogenomics, Department of Cancer Biology, The Gujarat Cancer & Research Institute, Ahmedabad, Gujarat, India; University of Texas MD Anderson Cancer Center, UNITED STATES

## Abstract

**Background & aim:**

Liver metastasis has been found to affect outcome in prostate, pancreatic and colorectal cancers, but its role in lung cancer is unclear. The 5 year survival rate remains extensively low owing to intrinsic resistance to conventional therapy which can be attributed to the genetic modulators involved in the pathogenesis of the disease. Thus, this study aims to generate a model for early diagnosis and timely treatment of liver metastasis in lung cancer patients.

**Methods:**

mRNA expression of 15 genes was quantified by real time PCR on lung cancer specimens with (n = 32) and without (n = 30) liver metastasis and their normal counterparts. Principal Component analysis, linear discriminant analysis and hierarchical clustering were conducted to obtain a predictive model. The accuracy of the models was tested by performing Receiver Operating Curve analysis.

**Results:**

The expression profile of all the 15 genes were subjected to PCA and LDA analysis and 5 models were generated. ROC curve analysis was performed for all the models and the individual genes. It was observed that out of the 15 genes only 8 genes showed significant sensitivity and specificity. Another model consisting of the selected eight genes was generated showing a specificity and sensitivity of 90.0 and 96.87 respectively (p <0.0001). Moreover, hierarchical clustering showed that tumors with a greater fold change lead to poor prognosis.

**Conclusion:**

Our study led to the generation of a concise, biologically relevant multi-gene panel that significantly and non-invasively predicts liver metastasis in lung cancer patients.

## Introduction

Lung cancer is the foremost reason for mortality and morbidity globally and its incidence is vastly increasing [1]. The high mortality rate can be attributed to late diagnosis and increased metastatic potential of the disease. In several cases, multiple metastases from primary lung cancer to different organs develop at a clinically early stage or at the time of diagnosis [2, 3]. Moreover, 30–40% patients with advanced lung cancer develop liver metastases resulting into an increased morbidity and low survival rate [4, 5]. The treatment for metastatic lung cancer clinically consists of systemic therapy encompassing cytotoxic and/or molecularly-targeted agents and palliative radiotherapy for symptomatic management. But the life expectancy of lung carcinoma patients depends on the extent of the disease and the response to chemotherapy [6]. However, currently no curative therapy exists for patients suffering from lung cancer liver metastasis and thus treatment and prevention is of utmost importance for the management of this disease [5, 7, 8].

An emerging field of cancer research is to discover therapy for carcinomas that develop drug resistance and form distant metastasis. Prognostic biomarkers are anticipated to be beneficial for prediction of the probable course of lung cancer liver metastases that prominently leads to the aggressiveness of the disease. Moreover, multiple biomarkers are known to be involved in the pathogenesis of the disease and thus the detection of these multiple prognostic biomarkers may increase the diagnostic sensitivity and specificity over the use of individual markers. Recently promising strategies for such biomarker discovery have been known that consists of microarray-based profiling at the DNA and mRNA levels, and also mass-spectrometry-based profiling at the protein and peptide levels [9].

Recent studies have focused on the identification of biological markers that may be helpful in the prediction of early recurrence and death in advanced stage lung cancer patients. A prognostic biomarker panel in lung cancer patients have also been developed using molecular sub-staging and oncogenic factors to improve risk stratification of the TNM staging system [10, 11]. Contradictorily newer methods for predicting lung cancer metastasis involves feature (gene) selection and classifier design. Feature selection identifies a subset of differentially-expressed genes that are potentially relevant in distinguishing the disease from the normal samples. However, one of the principal difficulties in investigating microarray classification and gene selection is the availability of only a small number of samples, compared to the large number of genes in a sample [12]. Besides, hierarchical clustering [13] is also one of the most commonly used approaches in microarray as well as gene expression studies. Conversely, hierarchical clustering (or any purely correlative technique) cannot alone provide a rational biological basis for disease classification [14]. Therefore, multivariate analysis comprising of principal component analysis (PCA) and linear discriminant analysis (LDA) is also conducted to reduce and obtain a linearity of the massive data reproduced [15].

This study aimed to acquire a definite model that could be used to predict liver metastasis in patients suffering from advanced stage lung carcinoma. Here, we examined the differential expression profile of a multi gene panel specific for liver metastasis that we obtained in our previous study published in Meta Gene [16] in primary lung cancer (PL; n = 30) and lung cancer with liver metastasis patients (ML; n = 32) and normal lung tissues (NL). The gene expression data acquired by real time PCR was subjected the data to multivariate analysis like PCA and LDA which ultimately led us to the generation of a specific model. We further investigated the potential of the model that we generated to predict the onset of liver metastasis by ROC curve analysis and found that out of the 5 models generated model 4 holds impressive potential for prediction of liver metastasis from primary lung cancer patients. Further, we studied the individual sensitivity, specificity and cut-off values of each gene that were implicated in the model.

## Methods

### Patients

Sampling was done during routine Fine Needle Aspiration Cytology (FNAC) procedure as a part of diagnostic workup carried out at The Gujarat Cancer & Research Institute. 30 specimens of primary lung cancer and 32 tissue specimens from patient histo-pathologically confirmed for liver metastasis were collected with prior consent. The median age of the patients was 60 years at diagnosis, ranging from 30 to 82. The study was approved by the Institutional ethics committee of The Gujarat Cancer and Research Institute and written consent was obtained from all the patients with the approval of the consent procedure from the ethics committee. Clinico-Pathological characteristics including tumor location, age, gender, habit, stage, and differentiation was noted in each case.

### Quantitative Real-time PCR

Tissue samples were stored in RNA later immediately after FNAC biopsy. Total RNA was isolated using RNeasy tissue kit (Qiagen 74106) according to the manufacturer’s instructions. RNA extracted from normal lung and liver (Agilent Technologies, USA & Clontech, Takara Bio Company, USA) was used as control for the study. RNA integrity was examined by gel-electrophoresis on 1% formaldehyde gel. The concentration of the isolated RNA was quantified with Qubit 3.0 Fluorometer (Invitrogen by Life Technologies,CA, USA). 1.0 μg total RNA was reverse transcribed to cDNA using the cDNA archive kit (Applied Biosystems–ABI; Cat no: 4368813) in 50μl reaction volume following manufacturer’s instructions. Real-time PCR was performed in a final volume of 20 μl containing 10 μl Brilliant III Ultra-Fast SYBR Green QPCR Master Mix (Agilent Technologies, USA), 0.5 μl (200 nM) each of the forward primer & reverse primer, and 2 μl cDNA. β-actin was used as a housekeeping gene in each set of experiment. The list of all the primers used for the study (CXCL12, CXCR4, CK7, CDH1, CTNNB1, CLDN4, HIF1A, MMP9, VEGFA, OPN, CDKN2A, TGFBR2, MUC16, TP53, CD44v6 and β-actin) are shown in Table 1. Quantitative PCR using Sybr Green chemistry was carried out in AriaMX (Agilent Biosystem) in a 96-well reaction plate format with at the following cycling conditions: 1 cycle of 3 min at 95°C for the initial denaturation step and 40 cycles of 5 s at 95°C for the denaturation step, 20 s at 60°C for the annealing and extension step. Melting curve analysis was performed following the amplification in order to ensure positive amplification of the target gene rather than non-specific products or primer dimmers. The relative fold change in expression was calculated using the ΔΔCT method. All experiments were performed in triplicate independently and average C_T_ value was used for further calculations.

**Table 1:** Sequences and primer sets used for real time PCR.

Gene	Forward Primers	Reverse Primers
CXCL12	5’ -AAGCCCGTCAGCCTGAGCTA-3’	5’ -TTAGCTTCGGGTCAATGCACAC-3’
CXCR4	5’-AATAAAATCTTCCTGCCCACC-3’	5’-CTGTACTTGTCCGTCATGCTTC-3’
CK7	5’-GACATCGAGATCGCCACCTAC-3’	5’-ATTGCTGCCCATGGTTCCC-3’
CDH1	5′-GACTCGTAACGACGTTGCAC-3′	5′ -GGTCAGTATCAGCCGCTTTC-3′
CTNNB1	5′-TGGATACCTCCCAAGTCCTG-3′	5′-CAGGGAACATAGCAGCTCGT-3′
CLDN4	5′-AGATGGGTGCCTCGCTCTAC-3′	5′-CCAGGGAAGAACAAAGCAGA-3′
HIF-1α	5’–ACAGCCTCACCAAACAGAGCAG-3’	5’–CGCTTTCTCTGAGCATTCTGCAAAGC-3’
VEGFA	5’-CTTGCCTTGCTGCTCTACC-3’	5’-CACACAGGATGGCTTGAAG-3’
MMP9	5'-GAGTGGCAGGGGGAAGATGC-3'	5'-CCTCAGGGCACTGCAGGATG-3'
p53	5′-CCGTGTTGGTTCATCCCTGTA-3′	5′-TTTTGGATTTTTAAGACAGAGTCTTTGTA-3′.
OPN	5′- ACTCGTCTCAGGC CAGTTG-3′	5′-CGTTGGACTTGGAAGG- 3′
CDKN2A	5’-CCCAACGCACCGAATAGT-3’	5’-GGGGATGTCTGAGGGACCTT-3’
TGFβR2	5’-GTAGCTCTGATGAGTGCAATGAC-3’	5’-CAGATATGGCAACTCCCAGTG-3’
MUC16	5’-CTGAGACCCCAACATCCTTG-3’	5’-GGTCACTAGCGTTCCATCAG-3’
β- actin	5’-TGACGTGGACATCCGCAAAG-3’	5’-CTGGAAGGTGGACAGCGAGG-3’

### Hierarchical clustering

To investigate whether the expression profile in the primary tumor can specifically identify metastatic status, hierarchical unsupervised clustering was performed using the hcluster method of R package “amap” and the plot was generated using the heatmap.2 function of “gplots” package. Absolute Pearson and Pearson distances were used to calculate gene and sample distances respectively and gene linkages were done using the Ward algorithm. Inter-study normalization was done with the Bioconductor package “inSilicoMerging” using an Empirical Bayes method.

### Principal component analysis

To obtain a reliable model for prediction of liver metastasis, data was analyzed statistically by applying Principal Component Analysis (PCA) method using SPSS 20.00 statistical software (Chicago, IL, USA). Data dimensionality was reduced by using an orthogonal transformation to convert correlated variables into uncorrelated variables, which are termed principal components. PCA has a lower or equal number of principal components compared to the number of original variables. PCA score was obtained using the following formulae:

PCAscore=C1V1+C2V2+C3V3+C4V4+C5V5+⋯+CnVn

where C1, C2, C3 … Cn are coefficients of each of the variables and V1, V2, V3 … Vn are the values of original variables

PCA score was generated for each individual patient. Multivariate Cox regression analysis was used to define the variables included in the PCA analysis. To scrutinize the performance of the new PCA-based method, the distribution of accuracy, specificity, and sensitivity was studied. For this, patient stratification process was simulated 100 times by resampling. The patients were divided into primary lung cancer set (30 patients, 48%) and liver metastatic test set (32 patients, 52%) groups. PCA was applied to the data sets to determine the coefficient of each variable, and a PCA score was generated for each patient. The data sets were then stratified into groups based on the mean value of the PCA scores. Accuracy, specificity, and sensitivity were calculated as follows:

Accuracy=(TP+TN)/(TN+TP+FP+FN)

Sensitivity=(TP)/(TP+FN)

Specificity=(TN)/(FP+TN)

where TP, TN, FP, and FN are true positive, true negative, false positive, and false negative, respectively.

Statistical analyses and the PCA algorithm were performed using SPSS 20.00 software and a p-value of < 0.05 was considered statistically significant [17].

### Linear discriminant analysis & receiver operating characteristics curve analysis

Linear Discriminant Analysis (LDA) was used to determine whether mRNA expression patterns could accurately discriminate liver metastasis in an independent data set. The accuracy of the predicted model was calculated using 1000 repetitions of a random partitioning process to regulate the number and proportion of false discoveries [18]. For diagnostic accuracy and discriminating metastatic tumor from primary tumor, a held-out test set from each database was utilized to evaluate the performance of each of the different classifiers. Receiver operator characteristics curves (ROC) were generated and AUCs of each classifier were calculated using MedCalc (Belgium, Europe). To understand the false positives and/or weaknesses of our classifiers, images frequently misclassified by the classifiers were also reviewed.

## Results

### Gene expression pattern in metastases versus those of primary tumors

**Fig 1:**
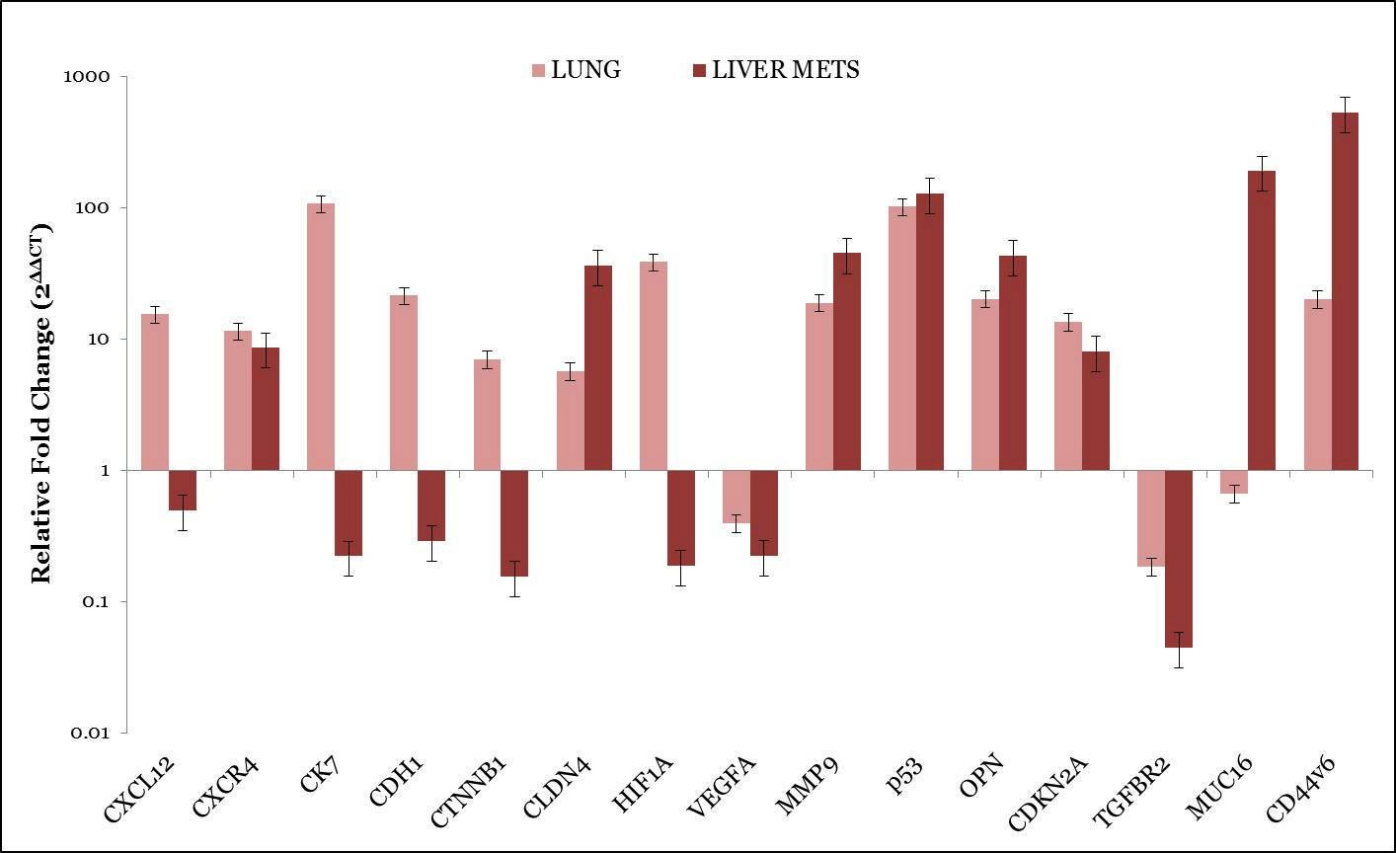
Gene expression in primary lung tumors, advanced stage lung cancer with liver metastasis, as well normal liver tissue were analyzed by quantitative RT-PCR.

In order to find a gene expression pattern that can discriminate metastatic tumors from primary tumors, differentially expressed genes between liver metastases and primary lung adenocarcinomas were identified as depicted in Fig 1. Among these genes, CXCL12, CXCR4, CK7, CDH1, CTNNB1, CLDN4, HIF1A, MMP9, CDKN2A, TP53, OPN and CD44v6 were upregulated (≥2 fold differently expressed) whereas VEGFA, TGFBR2 and MUC16 were downregulated (< 2fold differently expressed) in primary tumors as compared to normal lung tissue. On the other hand CXCR4, CLDN4, MMP9, OPN, TP53, CDKN2A, MUC16 and CD44v6 were upregulated (≥2 fold differently expressed) and CXCL12, CK7, CDH1, CTNNB1, HIF1A, VEGFA and TGFBR2 were downregulated (< 2 differently expressed) in lung cancer with liver metastasis as compared to primary tumor and Normal lung tissue. When we compared the expression of the above mentioned genes between both the cohorts–primary and metastatic we found that CLDN4, MMP9, OPN, CDKN2A, TP53, MUC16 and CD44v6 showed significant up-regulation and VEGFA and TGFBR2 showed significant downregulation in liver metastatic patients as compared to primary lung cancer. CXCR4, CK7, CDH1, CTNNB1 and HIF1A showed significant upregulation in primary lung cancer as compared to liver metastasis. The detailed gene expression fold changes for all the patients included in the study are provided in the S1 Table and S2 Table for metastasis and primary tumor respectively.

### Hierarchical clustering

**Fig 2:**
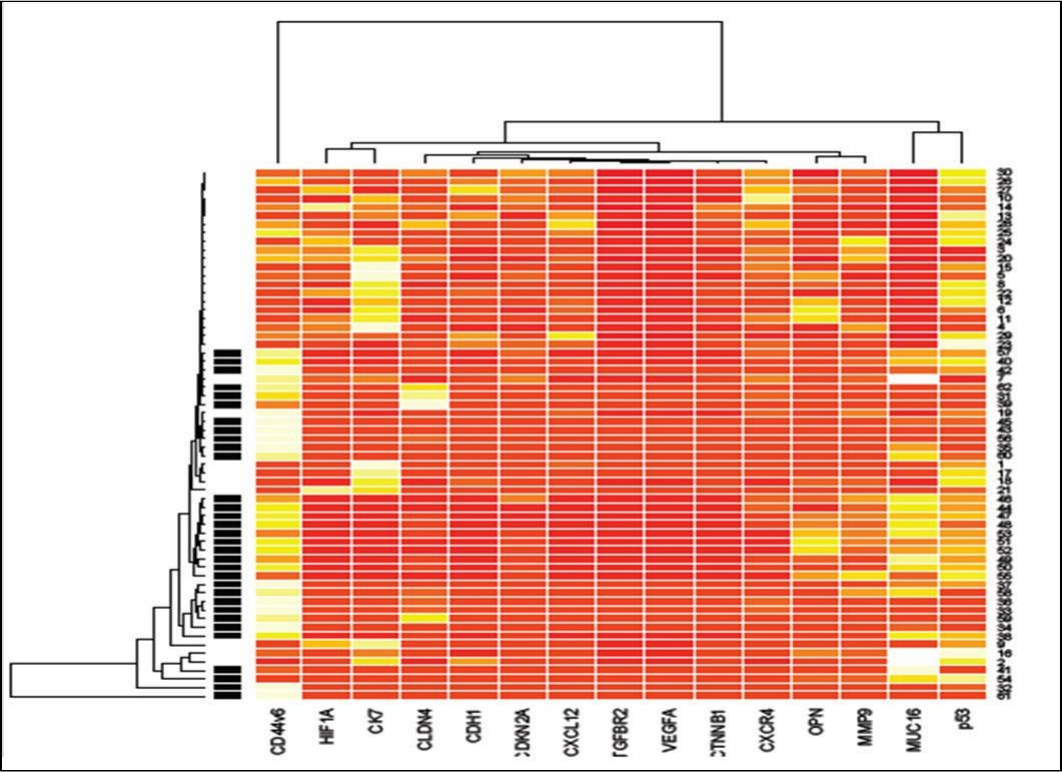
Unsupervised hierarchical clustering of 30 primary lung cancer samples and 32 advanced stage lung cancer liver metastatic samples based on 15 differentially expressed genes at a false discovery ratio level of 0.05. Tumor identification appears at the top of the figure and each column represents gene expression of a single tumor. UniGene cluster ID or gene ID or ORESTES is shown in each row. The colored bar indicates the variation in gene expression in target samples as compared to reference cells i.e., red, more expressed and white, less expressed in target samples. The black lines of the dendrogram stand for the support for each clustering. The metric used was Euclidean distance, with complete linkage for distance between clusters.

A query was generated whether the selected 15 genes would be useful in classifying primary tumors into groups that have different potential to develop liver metastasis or not. This could only be anticipated to happen if the gene expression profile associated with metastasis is already present in a subset of cells in the primary tumors. The expression of the 15 genes was therefore used in hierarchical clustering to classify a group of 30 non-metastatic tumors as depicted in Fig 2. The tumors were clustered into two distinct groups, based on their expression profile in primary and metastatic tissue as highly correlating or not correlating with each other. We predicted that the tumors with a greater fold change in gene expression profile would have a worse prognosis may be due to disease progression to metastasis. In patients with primary lung tumors (PL) where follow-up data were available, 50% primary tumors developed metastases, whereas from the other 50% only 25% developed metastases.

### Construction of a Gene Expression–Based Outcome Predictor Model and Analysis of Sensitivity and Specificity

**Fig 3:**
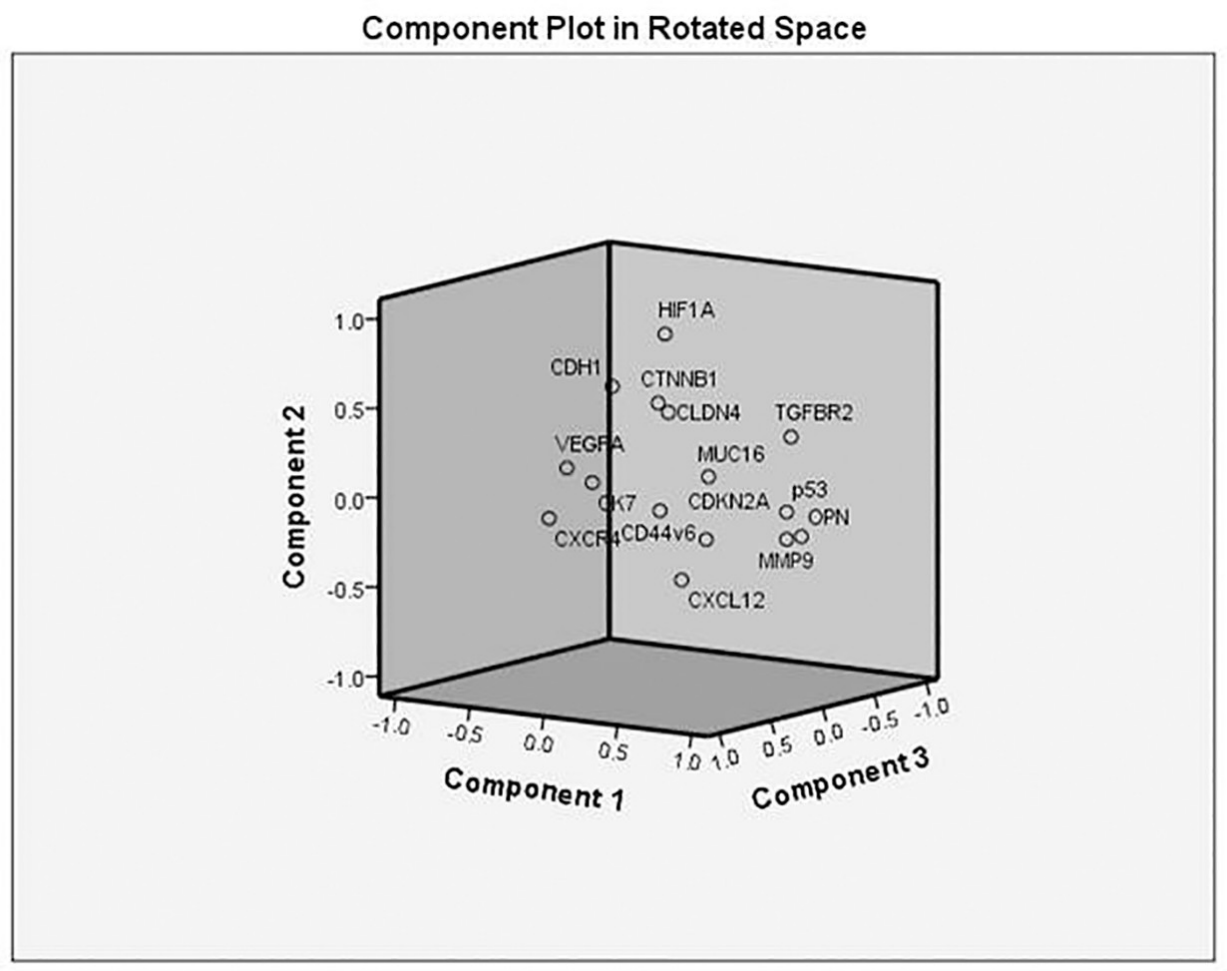
Principal component analysis (PCA) indicative of the variability of the gene expression data within each of the two patient groups. The x, y, and z axes are the first, second, and third components that together capture most of the variability.

In all five PCA models for lung cancer were constructed using the marker expression as variables (CXCL12, CXCR4, CK7, CDH1, CTNNB1, CLDN4, HIF1A, VEGFA, MMP9, MUC16, TGFBR2, TP53, OPN, CDKN2A, and CD44v6) as shown in Fig 3. The PCA scores plot showed that the primary cancer group and metastatic group samples were scattered into different regions in Model 4. Further the model was subjected to Linear Discriminant analysis and a linear equation for the proposed model was obtained.

Equation:(CoefficientofGene1*expressionofGene1)+⋯……….(CoefficientofGeneN*expressionofGeneN)

**Fig 4:**
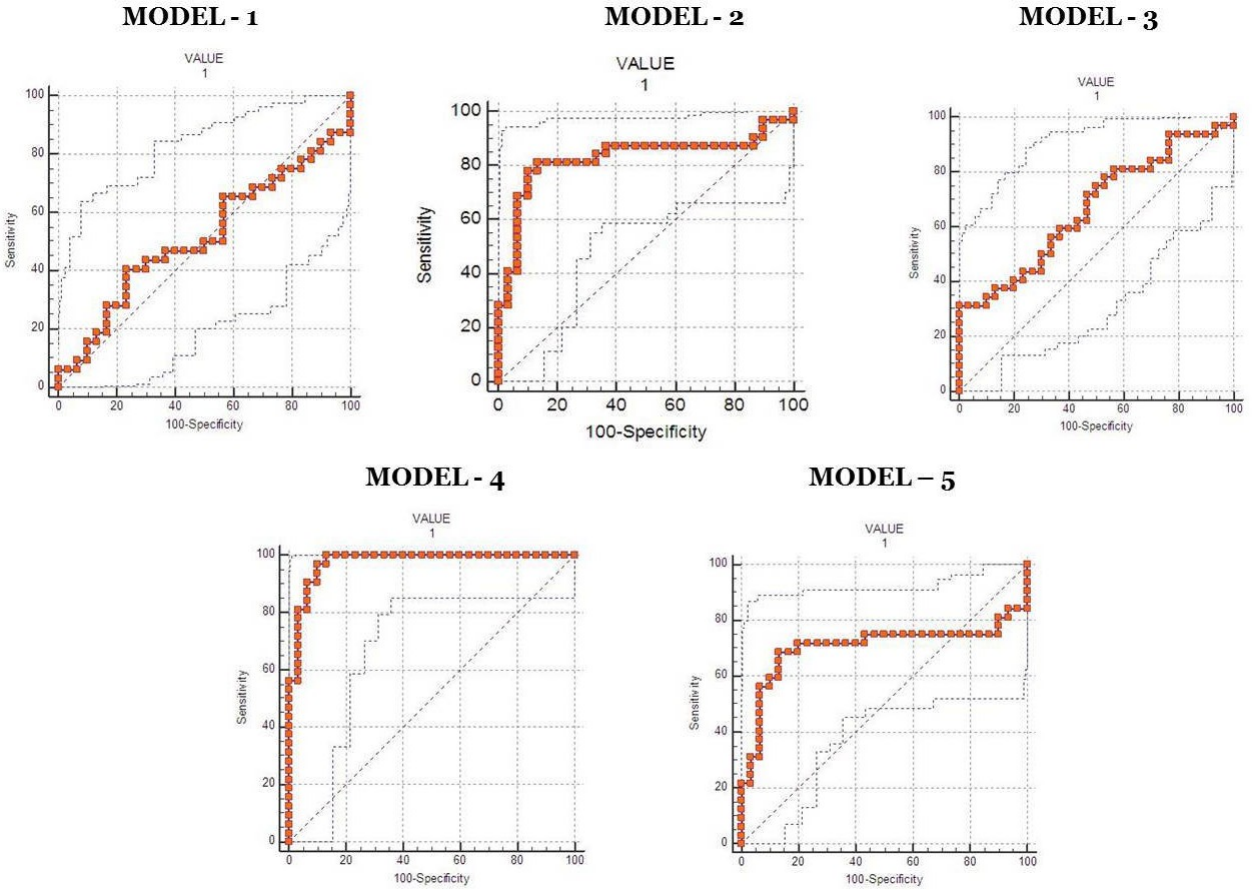
Receiver Operating Characteristic Curve analysis comparing the various predictive models based on the 15 gene panel.

**Fig 5:**
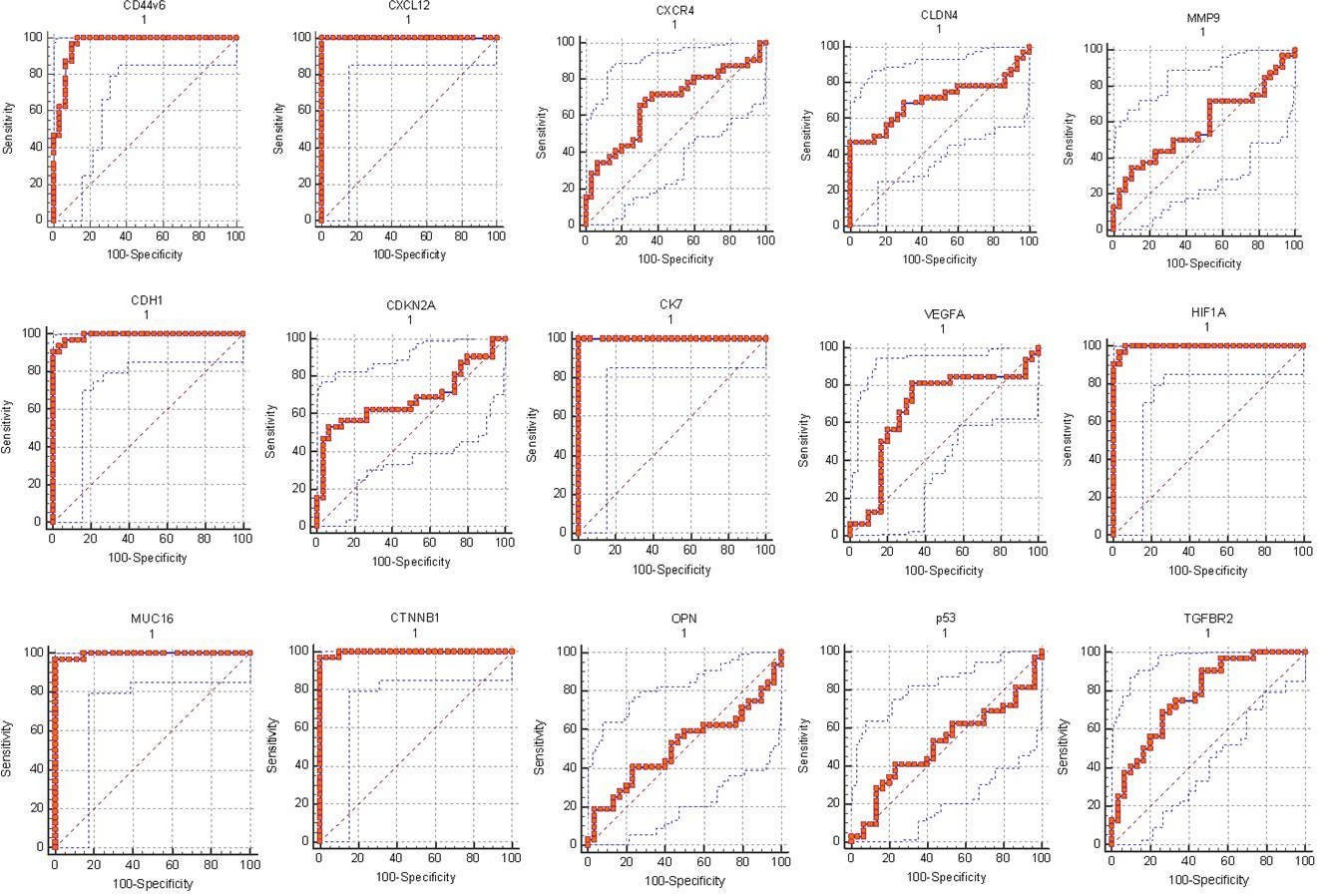
Receiver Operating Characteristic Curve analysis of the individual 15 genes in patients with primary lung cancer with and without liver metastasis.

**Fig 6:**
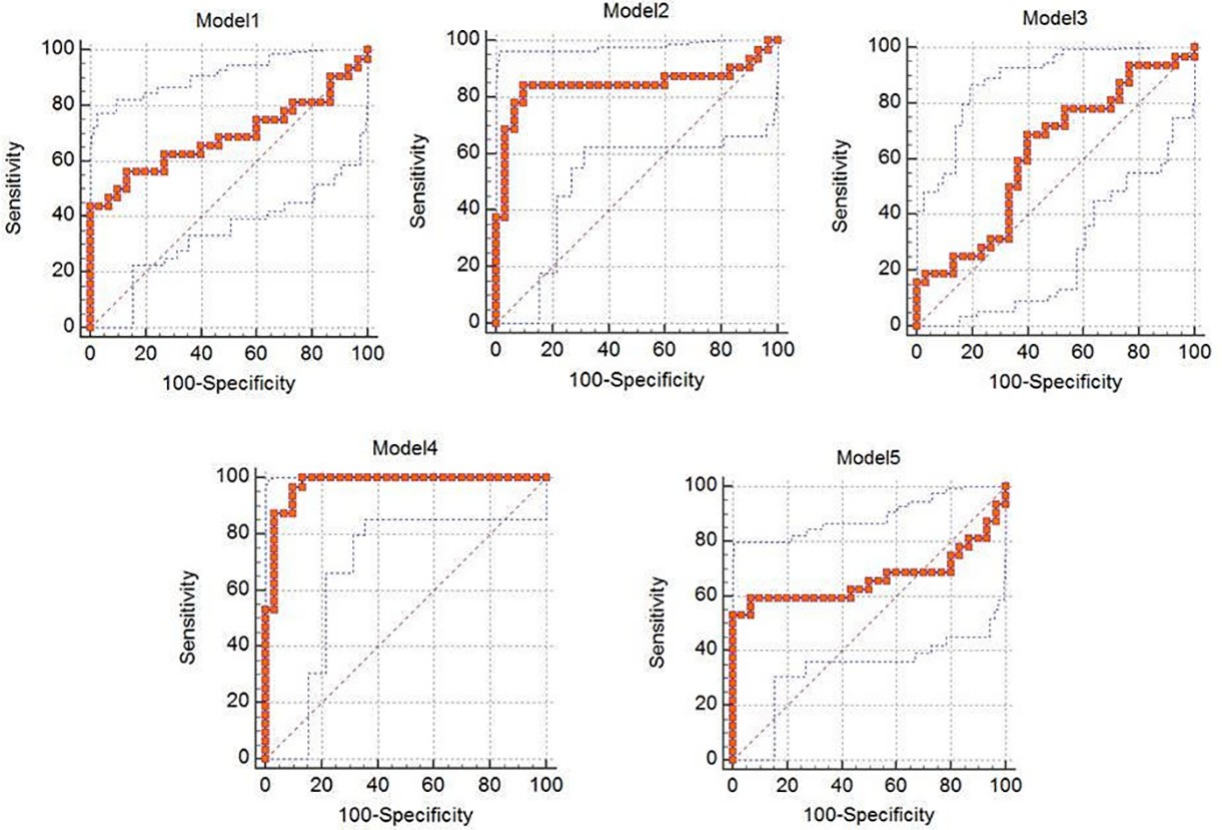
Receiver Operating Characteristic Curve analysis comparing the various prediction models based on the 8 gene panel after filtering the non-correlating genes.

ROC analysis was performed for all the 5 proposed models and the details are mentioned in Table 2. ROC analysis, which was performed, using the values, determined by the PCA model, and confirmed the robustness of the PCA model 4. Area under the curve (AUC) for model-4 was 0.975 with specificity and sensitivity of 90% and 96.87% respectively (Fig 4), which demonstrated a good discriminative value for lung cancer liver metastasis. In order to determine if the multigene signature has any practical application, we performed ROC analysis for individual genes as seen in Fig 5 and the sensitivity, specificity & AUC are mentioned in Table 3. From the ROC curve analysis of individual genes we further speculated that a more precise model for prediction of lung liver metastasis could be formed with the 8 gene (CXCL12, CK7, CDH1, CD44v6, HIF1A, MUC16, CTNNB1 and TGFBR2) algorithm as described in Fig 6 and Table 4. Moreover, it was observed that Model– 4 came out as the best model that can be used for predicting liver metastasis with the highest AUC of 0.975 a Specificity and Sensitivity of 90% and 96.87% respectively showing good discriminative ability between primary and metastatic tumors.

**Table 2:** ROC curve details for all the PCA models.

Model Name	Associated Criteria	Sensitivity	Specificity	Significance P (area = 0.5)	Youden Index J	Area under the curve	95% CI
Model 1	>26.93393745	40.63	76.67	0.8461	0.1729	0.515	0.384 to 0.644
Model 2	≤8.574554323	78.12	90.00	<0.0001	0.6813	0.827	0.710 to 0.911
Model 3	≤3.151672989	31.25	100.0	0.0159	0.3125	0.667	0.535 to 0.781
Model 4	>18.46076597	96.87	90.00	<0.0001	0.8688	0.975	0.899 to 0.998
Model 5	>11.93832043	68.75	86.67	0.0067	0.5542	0.703	0.574 to 0.812

**Table 3:** ROC curve analysis of the individual genes.

Model Name	Associated Criteria	Sensitivity	Specificity	Significance P (area = 0.5)	Youden Index J	Area under the curve	95% CI
CD44v6	>27.66519140	96.87	90	<0.0001	0.8688	0.965	0.884 to 0.995
CXCL12	≤1.578258295	100	100	<0.0001	1	1	0.942 to 1.000
CXCR4	≤6.535661813	65.62	70	0.0147	0.3563	0.671	0.540 to 0.785
CDH1	≤0.901250463	90.62	100	<0.0001	0.9063	0.992	0.927 to 1.000
CDKN2A	≤2.060984041	53.13	93.33	0.0103	0.4646	0.682	0.552 to 0.795
CK7	≤0.649169294	100	100	<0.0001	1	1	0.942 to 1.000
CLDN4	>17.28761168	46.88	100	0.0048	0.4688	0.699	0.569 to 0.809
CTNNB1	≤0.698984967	96.87	100	<0.0001	0.9688	0.997	0.936 to 1.000
HIF1A	≤0.766664172	96.87	96.67	<0.0001	0.9354	0.996	0.934 to 1.000
MMP9	>40.36407708	34.38	90	0.2545	0.2438	0.584	0.452 to 0.708
MUC16	>1.972465409	96.87	100	<0.0001	0.9688	0.995	0.930 to 1.000
OPN	>13.70534430	40.63	76.67	0.8689	0.1729	0.512	0.382 to 0.642
TP53	>57.81345285	40.63	76.67	0.8904	0.1729	0.510	0.380 to 0.640
TGFBR2	≤0.105112052	90.62	53.33	<0.0001	0.4396	0.774	0.650 to 0.871
VEGFA	≤0.23815950	81.25	66.67	0.0159	0.4792	0.678	0.547 to 0.791

**Table 4:** ROC curve details for the final shortlisted PCA models.

Model Name	Associated Criteria	Sensitivity	Specificity	Significance P (area = 0.5)	Youden Index J	Area under the curve	95% CI
Model 1	<-3.16089968	43.75	100.0	0.0077	0.4375	0.689	0.558 to 0.800
Model 2	<-3.96498514	84.37	90.00	<0.0001	0.7438	0.845	0.730 to 0.924
Model 3	≤13.19357517	68.75	60.00	0.1096	0.2875	0.617	0.484 to 0.737
Model 4	>15.25019492	96.87	90.00	<0.0001	0.8688	0.975	0.899 to 0.998
Model 5	>23.61467974	53.13	100.0	0.0317	0.5313	0.665	0.533 to 0.780

## Discussion

Recent studies have stated that different tumors metastasize to preferred secondary sites, depending on organ-susceptibility to specific cells but the molecular basis of organ tropism, one of the foremost hallmarks of cancer, still remains unclear. In 1889, Stephen Paget proposed the ‘seed and soil’ hypothesis. This theory clearly states that the molecular interaction between metastatic cells (seeds) and stromal microenvironment (soil) plays a critical role in the development of the multi-complex metastatic cascade [19, 20]. During the complex cascade the tumor cells from the primary site disseminate and enter into the circulation from where they colonize into the secondary organs to develop distant metastatic tumors. The cancer cells when homing to the specific target organ for the metastatic cascade to be successful must interact with the distant microenvironment which might initiate the activation or in activation of the genes in a coordinated fashion [5, 21]. These changes reflect the interaction between tumor cells as well as the host cells in the ‘microenvironment’ of the target organ.

High–throughput technologies are capable of extensive analysis of mRNA, miRNA as well as protein expression profiles on a larger scale with higher sensitivity as compared to the conventional techniques. Recent studies of lung cancer metastasis, with clinical genomic or proteomic approaches have identified subgroups of tumors that differ in terms of tumor type, histologic subclass, and patient survival allowing prediction of regional Lymph Node (LN) metastasis but not distant metastasis [22]. The study concluded the possibility that gene signatures from mRNA expression profiling can predict early metastasis and clinical outcome occurring in lung adenocarcinoma patients. However, to be used in clinical practice such as in preoperative chemotherapy, biopsy specimens should be collected from the primary tumor for gene expression analysis. Successful application of such a technique has already been reported by Borczuk et al. [23].

Numerous multi-gene signatures from mRNA expression profiling are capable of predicting LN metastasis in various primary malignancies together with the extent of the metastasis in distant sites. Contradictorily, the site of metastasis such as brain, bone, liver and lung cannot be identified with these multi gene signatures alone because of the heterogeneity amongst the primary and metastatic tumor. Even though mRNA expression panels are established from microarray data sets some individual genes in the panel might not be useful for predicting metastasis because of the complexity of most types of cancer and the compound nature of gene functions. Thus, it is desirable that gene panels representing the characteristic gene expression profiles of metastasis are selected and their interactions interpreted as a whole. Furthermore, because our ultimate goal is prediction of liver metastasis from primary lung cancer for new patients with the model developed from the fixed data, the model’s robustness is essential for the classification algorithm. Owing to this reason, we categorically used a modified model constructed from a series of known classification algorithms [24].

In this study we used a previously established multi-gene panel for generating a model specific for prediction of liver metastasis. We generated the model using PCA and LDA and further calculated the accuracy of the model generated by ROC curve analysis that showed accuracy greater than 85%. Moreover, the clustering data also showed a significance (p<0.0001) amongst the gene expression patterns of the different genes and their association with primary as well as metastatic tumor. Thus, the eight gene panel (CXCL12, CK7, CDH1, CTNNB1, CD44v6, MUC16, TGFBR2 and HIF1A) can be a highly significant predictor of liver metastasis outcome independent of the standard prognostic criteria. The eight-gene panel obtained after profiling genes across multiple pathways robustly predicted clinical outcome. Additionally, the ability of this panel to accurately predict recurrence in the liver independent of stage of the primary tumor is likely to be a useful enhancement to routine staging.

The genes identified in this study are likely not conventional tumor-derived cancer biomarkers but rather reflect subtle alterations in gene expression aiding as a systemic response to disease, probably acting to maintain homeostasis30 or facilitating disease pathology [25]. Thus, for example, one of the biomarker genes identified in this study, the chemokine stromal cell-derived factor-1 (SDF-1)/CXCL12 represents a natural ligand for the chemokine receptor CXCR4. Moreover, CXCL12 possesses angiogenic properties and is known to be involved in the outgrowth and metastasis of CXCR4-expressing tumors [26]. Furthermore, this axis has also been recognized as a prognostic marker in several tumors and preclinical models; signifying that metastasis is mediated by CXCR4 activation and migration of cancer cells towards CXCL12 expressing organs [27]. Another biomarker of interest in carcinogenesis, E- cadherin (CDH1) a single pass transmembrane protein is involved in epithelial to mesenchymal transitions (EMT) resulting in tumor progression and transition to a more motile and invasive phenotype [28, 29]. CTNNB1 also known as β-catenin plays a vital role in the regulation of the E-cadherin-catenin cell adhesion complex and further functions in growth signalling events, independently of the cadherin-catenin complex [30]. Recent studies have revealed that nuclear accumulation of β-catenin during invasive stages of primary tumor may lead to significant upregulation of this gene and has been significantly associated with liver metastasis from colorectal carcinoma [31].

Mucin -16 (MUC16) also known as CA-125 is the largest membrane associated mucin which possesses a single transmembrane domain and is a repeating peptide epitope [32]. It promotes cancer cell proliferation, causes inhibition of the anti-cancer immune responses and is reported to have been upregulated in multiple malignancies [33, 34]. Additionally CK-7 also known as Keratin-7 (KRT-7) and Transforming growth Factor beta receptor 2 (TGFBR2) are known to have an active participation in metastasis promotion and progression [35]. HIF1α also known as Hypoxia Inducing Factor 1α plays an important role in the formation of liver metastasis. It has been reported that HIF1A overexpression enhances ZEB1 transactivity by binding to its promoter leading to a loss in E-cadherin and increased cell invasion and migration [36]. Finally Cluster of Differentiation 44 variant 6 (CD44v6) is known to be a major player in shedding off cells from the primary tumor to the distant metastatic site. It has been observed that cytokines hepatocyte growth factor (HGF), osteopontin (OPN), and stromal-derived factor 1α (SDF-1), secreted from tumor associated cells, increase CD44v6 expression by activating the Wnt/β-catenin pathway, which promotes migration and metastasis [37]. Subsequently the biomarkers shortlisted for the panel are in some way or other involved in the pathogenesis of liver metastasis. Therefore, the generated model could aid in the early diagnosis of liver metastasis.

In conclusion, using a multiplexed, molecularly driven approach, we have identified a panel comprising CXCL12, CK7, CDH1, CTNNB1, CD44v6, MUC16, TGFBR2 and HIF1A that can predict recurrence in the liver independent of conventional prognostic criteria and identify patients with lung cancer who will develop liver metastasis despite undergoing definitive surgery and/or treatment. Increasing numbers of alterations in these genes predict poorer prognosis. Additional validation study of this panel prospectively in larger set and alternate sample source e.g. Cell free nucleic acid, exosomes is necessary to better characterize its ability to identify patients at higher risk in a non-invasive way. Hence, this multi-gene panel and their associated pathways may serve as promising outcome predictors and potential therapeutic targets in lung cancer patients with liver metastasis.

## Supporting information

S1 Table Gene expression analysis of 15 genes for all the metastatic patients by quantitative RT-PCR. (XLSX)

S2 Table Gene expression analysis of 15 genes for all the primary lung tumor patients by quantitative RT-PCR. (XLSX)
